# Worldwide Endemicity of a Multidrug-Resistant *Staphylococcus capitis* Clone Involved in Neonatal Sepsis

**DOI:** 10.3201/eid2303.160833

**Published:** 2017-03

**Authors:** Marine Butin, Patricia Martins-Simões, Jean-Philippe Rasigade, Jean-Charles Picaud, Frédéric Laurent

**Affiliations:** Hospices Civils de Lyon, Lyon, France (M. Butin, P. Martins-Simões, J-P. Rasigade, J-C. Picaud, F. Laurent);; INSERM, Lyon (M. Butin, P. Martins-Simões, J-P. Rasigade, F. Laurent);; Claude Bernard University Lyon 1, Villeurbanne, France (J-P. Rasigade, J-C. Picaud, F. Laurent)

**Keywords:** Staphylococcus capitis, Staphylococci, clone NRCS-A, multidrug-resistant, endemic, worldwide, neonatal sepsis, neonatal intensive care units, epidemiologic relevance, bacteria, antimicrobial resistance

## Abstract

A multidrug-resistant *Staphylococcus capitis* clone, NRCS-A, has been isolated from neonatal intensive care units in 17 countries throughout the world. *S. capitis* NRCS-A prevalence is high in some neonatal intensive care units in France. These data highlight the worldwide endemicity and epidemiologic relevance of this multidrug-resistant, coagulase-negative staphylococci clone.

Preterm birth is the world’s leading cause of death before 5 years of age (*1*). Neonatal sepsis, mostly due to coagulase-negative staphylococci, occurs frequently in neonatal intensive care units, especially in very low birthweight preterm infants (*2*). Cases and series of neonatal sepsis involving *Staphylococcus capitis* have been reported in different countries (*3*) and were initially considered unrelated epidemic bursts. More recently, we detected a single multidrug-resistant clone of *S. capitis*, designated as the NRCS-A clone and characterized by a specific pulsed-field gel electrophoresis (PFGE) pattern, in several neonatal intensive care units (NICUs) in France, Belgium, the United Kingdom, and Australia (*4**,**5*). The clonality of the strains was confirmed by PFGE, multilocus sequence typing–like analysis, and whole-genome sequencing. We also showed that all NRCS-A isolates exhibited a decreased susceptibility to all of the antimicrobial agents frequently used in NICUs, namely β-lactams, aminoglycosides, and vancomycin (*5*). Furthermore, a recent study showed that *S. capitis* NRCS-A–associated sepsis constitutes an independent risk factor for severe illness in neonates (*6*).

We suspected that the initial report of NRCS-A dissemination in NICUs from 4 distant countries was only the tip of the iceberg and that the spread of NRCS-A strains was much wider than expected. To determine the extent of NRCS-A dissemination, we asked microbiologic laboratories worldwide to send us methicillin-resistant *S. capitis* strains isolated from blood cultures of neonates. These isolates were identified by matrix-assisted laser desorption/ionization time-of-flight mass spectrometry and subjected to PFGE using the *Sma*I restriction enzyme as previously described (*7*). NRCS-A’s characteristic PFGE pattern was found for 154 strains isolated between 1994 and 2015 in 34 NICUs from 17 countries: Australia, Belgium, Brazil, Canada, Czech Republic, Denmark, Finland, France, Germany, the Netherlands, New Zealand, Norway, South Korea, Switzerland, Taiwan, the United Kingdom, and the United States.

Retrospective, laboratory-based epidemiologic investigations to estimate the prevalence of NRCS-A strains in NICUs could not be performed on the same worldwide scale, so we conducted such a study in France. Results collected from 47 of the 57 NICUs in France during 2014 indicated that only 4 NICUs were free of NRCS-A. In the 43 other NICUs, NRCS-A strains accounted for up to 46% of all cases of positive cultures of blood from neonates (median 13%, interquartile range 10%–20%) and represented 19% of all coagulase-negative staphylococci strains isolated from the blood cultures of neonates.

Taken together, these data unquestionably demonstrate the unusual worldwide endemicity of the multidrug-resistant NRCS-A clone in NICUs. In addition, the epidemiologic data from France highlight the propensity of NRCS-A to invade and settle in most NICUs on a national scale. Once endemic in a NICU, NRCS-A strains expose infected neonates to a risk of therapeutic failure because treatment of neonatal sepsis involving methicillin-resistant coagulase-negative staphylococci is usually based on vancomycin and aminoglycosides, to which NRCS-A isolates are not susceptible (*3**–**5*).

A thorough investigation of the determinants of the worldwide spread of NRCS-A is urgently needed to unravel the dissemination routes and reservoirs of this multidrug-resistant clone and to succeed in managing and controlling its diffusion. The risk of vancomycin treatment failure warrants an investigation of alternate antimicrobial stewardship strategies, in particular linezolid, daptomycin, and ceftarolin, to treat NRCS-A–associated neonatal sepsis.

## References

[1] Liu L, Oza S, Hogan D, Perin J, Rudan I, Lawn JE, et al. Global, regional, and national causes of child mortality in 2000-13, with projections to inform post-2015 priorities: an updated systematic analysis. Lancet. 2015;385:430–40. 10.1016/S0140-6736(14)61698-6 25280870

[2] Boghossian NS, Page GP, Bell EF, Stoll BJ, Murray JC, Cotten CM, et al.; Eunice Kennedy Shriver National Institute of Child Health and Human Development Neonatal Research Network. Late-onset sepsis in very low birth weight infants from singleton and multiple-gestation births. J Pediatr. 2013;162:1120–4, 1124.e1. 10.1016/j.jpeds.2012.11.089 PMC3633723 23324523

[3] Van Der Zwet WC, Debets-Ossenkopp YJ, Reinders E, Kapi M, Savelkoul PH, Van Elburg RM, et al. Nosocomial spread of a *Staphylococcus capitis* strain with heteroresistance to vancomycin in a neonatal intensive care unit. J Clin Microbiol. 2002;40:2520–5. 10.1128/JCM.40.7.2520-2525.2002 12089273 PMC120592

[4] Rasigade J-P, Raulin O, Picaud J-C, Tellini C, Bes M, Grando J, et al. Methicillin-resistant *Staphylococcus capitis* with reduced vancomycin susceptibility causes late-onset sepsis in intensive care neonates. PLoS One. 2012;7:e31548. 10.1371/journal.pone.0031548 22348102 PMC3279402

[5] Butin M, Rasigade J-P, Martins-Simões P, Meugnier H, Lemriss H, Goering RV, et al. Wide geographical dissemination of the multiresistant *Staphylococcus capitis* NRCS-A clone in neonatal intensive-care units. Clin Microbiol Infect. 2016;22:46–52. 10.1016/j.cmi.2015.09.008 26404028

[6] Ben Said M, Hays S, Bonfils M, Jourdes E, Rasigade JP, Laurent F, et al. Late-onset sepsis due to *Staphylococcus capitis* ‘neonatalis’ in low-birthweight infants: a new entity? J Hosp Infect. 2016;94:95–8. 10.1016/j.jhin.2016.06.008 27424947

[7] Goering RV. Molecular epidemiology of nosocomial infection: analysis of chromosomal restriction fragment patterns by pulsed-field gel electrophoresis. Infect Control Hosp Epidemiol. 1993;14:595–600. 10.2307/30105130 7901269

